# Efficacy of combination colonoscopy using modified cap-assisted and water-exchange colonoscopy with prone position for detection of colorectal adenomas

**DOI:** 10.1097/MD.0000000000031271

**Published:** 2022-11-11

**Authors:** Jihwan Ko, Hyung Wook Kim, Dae Hwan Kang, Cheol Woong Choi, Seong Ho Han, Byung Gu Ko

**Affiliations:** a Health Promotion Center, Baekyang Jeil Internal Medicine Clinic, Busan, Korea; b Department of Internal Medicine, Pusan National University School of Medicine and Research Institute for Convergence of Biomedical Science and Technology, Pusan National University Yangsan Hospital, Yangsan, Korea.

## Abstract

The efficacy of cap-assisted and water-exchange colonoscopy, individually or in combination for adenoma detection is well documented. Moreover, prone positioning colonoscopy may also improve adenoma detection by decreasing loop formation. However, the efficacy of triple-combination colonoscopy using the above methods for adenoma detection is unclear. This study aimed to compare the effectiveness of combining modified cap-assisted and water-exchange colonoscopy with prone position (CWP) and conventional colonoscopy (CC) for adenoma detection.

A total of 746 patients who underwent either CWP or CC, performed by 2 board-certified gastroenterologists between December 2019 and March 2020, were investigated retrospectively. Cap-assisted colonoscopy was modified using hooking and dragging maneuver. We evaluated the polyp detection rate (PDR), adenoma detection rate (ADR), and the mean number of adenomas detected per procedure (MAP).

There was no significant difference in sex, age, the indication of colonoscopy and quality of bowel preparation between the 2 groups. PDR, ADR, and proximal colon MAP were significantly higher in the CWP group than in the CC group (PDR: 84.9% vs 59.8%, *P *< .01; ADR: 70.1%, vs 49.2%, *P* < .01; proximal colon MAP: 1.24 vs 0.55, *P* < .01).

CWP is more effective than CC for PDR, ADR, and proximal colon MAP. Although it may facilitate adenoma detection, further studies assessing the synergistic or complementary effects of combining these methods are needed.

## 1. Introduction

Colorectal cancer is the third leading cause of new cancer cases and the second leading cause of cancer deaths worldwide.^[1]^ Screening for colorectal cancer using colonoscopy has several advantages, including highly sensitive detection, and a single-step diagnosis and treatment of cancer or precancerous lesions.^[2]^ In addition, several studies have reported the efficacy of screening colonoscopy in preventing the incidence and deaths from colorectal cancer.^[3–6]^ However, despite the many advantages of screening colonoscopy, it has a drawback–detecting cancer and precancerous lesions using colonoscopy is operator dependent.^[7,8]^

Among many quality indicators for monitoring operator dependency, the adenoma detection rate (ADR) and the mean number of adenomas detected per procedure (MAP) are the most sensitive indicators of the quality of colonoscopy.^[9–11]^ Many studies have focused on the improvement of these quality indicators, and new techniques have been developed, such as cap-assisted colonoscopy,^[12]^ water-exchange colonoscopy,^[13]^ and a combination of the above 2 methods.^[10]^ Cap-assisted colonoscopy involves a transparent hood fitted to the tip of the colonoscope and allows better mucosal exposure, particularly in the regions behind the proximal aspect of the haustral folds; it also decreases the cecal intubation time. Water-exchange colonoscopy provides the advantage of additional cleansing and may aid in optimizing the mucosal examination.^[14]^

Combining the aforementioned methods can assist endoscopists in each phase of the colonoscopy–insertion, inspection, and intervention.^[10]^ Thus, devising a method that can increase ADR and MAP without using complicated devices, such as Endocuff, Endocuff-Vision, or Endorings,^[15]^ which cannot be applied in clinical settings because of the restrictions of the National Health Insurance system in Korea, and determining whether there is any synergistic effect with the simultaneous use of several methods are of vital importance. The addition of prone positioning to the combination of cap-assisted and water-exchange colonoscopy may prevent loop formation by redistributing abdominal pressure.^[16]^ Therefore, we aimed to compare ADR between the conventional colonoscopy (CC) and combination colonoscopy using modified cap-assisted and water-exchange colonoscopy with prone position (CWP).

## 2. Materials and Methods

### 2.1. Study design

This single-center, retrospective, case-control study was conducted at Baekyang Jeil Internal Medicine Clinic, Busan, South Korea. All procedures performed in studies involving human participants were performed in accordance with the ethical standards of the institutional and/or national research committee and with the 1964 Helsinki Declaration and its later amendments or comparable ethical standards. This study was approved by the ethics committee of the Institutional Review Board of Pusan National University, Yangsan Hospital (Institutional Review Board number: 05-2020-126). Informed consent was obtained from all participants included in the study.

### 2.2. Patients

**Figure 1.**
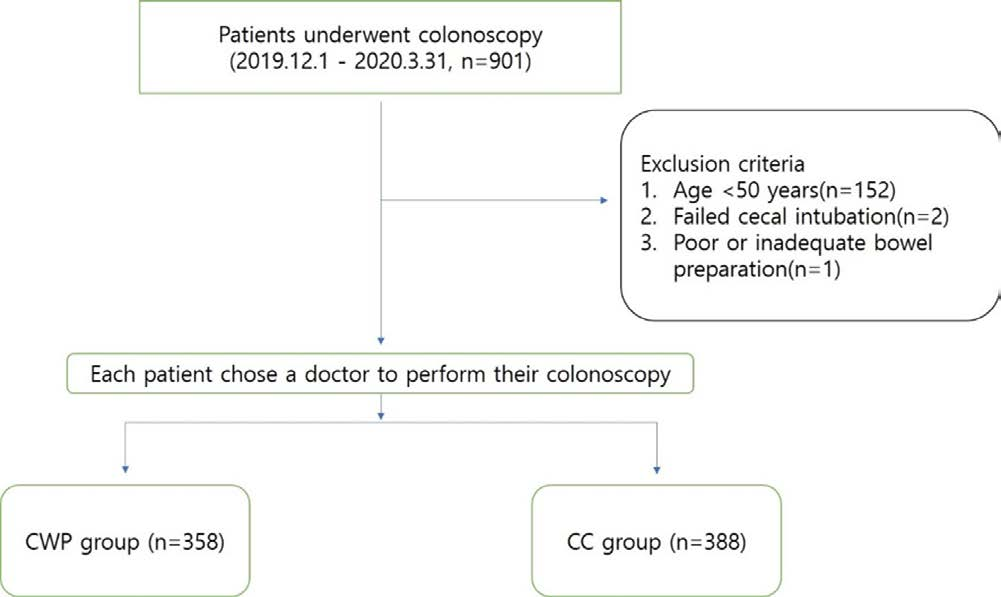
Flow chart of the participants. CC = conventional colonoscopy, CWP = Combination of modified cap-assisted and water-exchange colonoscopy with the patient in the prone position.

Between December 2019 and March 2020, a total of 901 patients underwent colonoscopies at our clinic. In total, 442 patients underwent CWP and 459 underwent CC. The procedures were performed by 2 board-certified gastroenterologists, J.K. and S.H.H. S.H.H. had 10 years of experience in CC, and J.K. had 3 years of experience in CC and 1 year in CWP. The exclusion criteria were age < 50 years, failed cecal intubation, poor or inadequate bowel preparation, previous colorectal resection, inflammatory bowel disease, colonic obstruction, and hereditary polyposis syndromes. Finally, among the 746 enrolled patients, 358 and 388 patients were included in the CWP and CC group, respectively (Fig. 1). The study groups were not randomized. Each patient chose a doctor to perform their colonoscopy. The indications for colonoscopy were for work-up of screening in 56.1%, whereas bowel symptoms, surveillance colonoscopy (i.e., patients with previous colonoscopy or colorectal polyps), and positive fecal occult blood test (FOBT) accounted for 9.7%, 20.5%, and 13.7%, respectively. Based on the indication for colonoscopy, the patients were divided into the following 2 groups: had a positive FOBT and were financially supported by the National Health Insurance System; and had a negative FOBT or did not undergo FOBT and were using their personal finances for the investigations related to polyps or cancer. In Korea, the National Health Insurance System encourages people aged over 50 years to undergo an FOBT annually; if the test result is positive, system provides financial support for the colonoscopy. Since the proportion of patients with positive FOBT could affect ADR,^[17]^ we compared the number of patients with positive FOBT for each group.

### 2.3. Endoscopic procedure

In this study, 2 board-certified gastroenterologists performed colonoscopies–1 had performed more than 10,000 CC procedures, and the other more than 3000 CWP procedures before the study period. No association had been reported between the procedural volume and the ADR; therefore, we assumed that the difference in the procedural volume between the 2 doctors would not affect ADR.^[18]^

Colonoscopies were performed after preparing the bowel with 2 L of polyethylene glycol plus an ascorbic acid solution (Coolprep, Taejun, Seoul, Korea; Readyfree, Intropharm Tech., Gyeonggi-do, Korea). Bowel preparation was evaluated and graded using the Aronchick scale.^[19]^ Colonoscopies were performed with EPK-i5000 (Pentax EPKi processor) and EC38-i10F colonoscopes (Pentax, Tokyo, Japan). A transparent cap was attached to the tip of the colonoscope (Finemedix Co. Ltd, Daegu, Korea). The modified cap-assisted colonoscopy, referred to as the “hooking and dragging method,” was performed by simultaneous hooking (gentle bending of the tip of the colonoscope towards the wall of the colon) and dragging (gentle retraction of the colonoscope dragging it down the multiple folds of the colon) the colonoscope down the colonic folds; for the procedure, a transparent hood was fitted onto the tip of the colonoscope.^[20]^

The colonoscopies were performed with the patients under midazolam-based conscious sedation. When polyps were detected, endoscopic observations and therapeutic interventions were carried out during the withdrawal phase. For both the procedures, the patients were initially made to lie in the left decubitus position. When the colonoscope reached the sigmoid colon, the patients in the CWP group repositioned themselves to a prone position and maintained the posture until the end of the procedure. During the colonoscopy, the patients used a hugging pillow to avoid chest compression; their oxygen saturation levels were monitored. The patients were sedated minimally because their cooperation was needed during the procedure for changing the position.

In the CWP group, the colonoscope with the waterjet system was inserted into the rectum with the air pump turned off. Residual air and fluid were immediately suctioned out of the colon, and warm water (at 37°C) was infused. The scope was advanced, and in each colonic segment, this exchange (removal of residual air and stool and infusion of sterile water) was performed. The appendiceal orifice was identified; water suction performed, and the air insufflated to facilitate the inspection and removal of the lesions. Upon the withdrawal of the scope, a transparent cap was used, and the haustral folds were flattened, to facilitate mucosal inspection. The air was insufflated to distend the lumen of the colon during scope insertion in the CC group. The water was used for irrigation only if bubbles or residual debris were encountered. The similarity and differences of the operations between 2 colonoscopies are summarized in Table 1.

**Table 1:** The similarity and differences of the operations between 2 colonoscopies.

	CWP	CC
Similarity	Starts with left decubitus position	
Differences	1.When the colonoscope reached the sigmoid colon, the patients repositioned themselves to a prone position 2.Hooking and dragging maneuver applied 3.Manual compression cannot be used when loop is formed	1.Keep left decubitus or supine position 2.Without hooking and dragging maneuver 3.Manual compression can be applied when loop is formed

CC = conventional colonoscopy, CWP = combining modified cap-assisted and water-exchange colonoscopy with prone position.

### 2.4. Polyps

Polyps with a diameter of ≥ 5 mm were removed by endoscopic mucosal resection or cold snare polypectomy. Polyps with a diameter of < 5 mm were removed with cold forceps polypectomy. All the removed polyps were examined pathologically, and the number of adenomas was determined. In this study, we defined the polyp detection rate (PDR) as the proportion of patients with at least 1 polyp, ADR as the proportion of patients with at least 1 adenoma, and MAP as the MAP (total number of adenomas/total number of procedures).

### 2.5. Outcome measures and statistical analysis

The raw data used for statistical analysis are shown in Supplement, http://links.lww.com/MD/H688. The primary outcome was the comparison of ADR between the CWP and CC groups. The secondary outcome was the comparison of PDR and MAP between these 2 groups. In addition, MAP in the proximal colon (proximal MAP) and the distal colon (distal MAP) were evaluated. The proximal colon was defined as the colon proximal to the splenic flexure, including the cecum, ascending colon, and transverse colon.

Statistical comparisons between the 2 groups were performed using the Mann-Whitney U-test, and ADR and PDR were analyzed using Fisher’s exact test. A *P* value of < 0.05 indicated statistical significance. The statistical analyses were performed using SPSS 26 (IBM Corp., Armonk, NY, USA).

## 3. Results

### 3.1. Patient characteristics

The baseline characteristics between the CWP and CC groups are summarized in Table 2. A total of 746 colonoscopies, including 358 CWP and 388 CC, were evaluated. The study population was composed predominantly of women (55.4%); the mean age was 62.9 years. For the indication of colonoscopy, 102 patients (13.7%) had positive FOBTs. The mean withdrawal time was 829.8 seconds, and the ratio of excellent bowel preparation was 76.8%. In the comparison between the 2 groups, there was no significant difference in sex, age, the indication of colonoscopy and quality of the bowel preparation except for the withdrawal time. Even though not intended, the cecal intubationt time. withdrawal time, and withdrawal time when polyp detection is zero was significantly longer in the CWP group than in the CC group (529.6 ± 367.4 vs 432.3 ± 288.1, *P* < .01; 950.70 ± 438.45 vs 718.15 ± 325.96, *P* < .01; 599.4 ± 139.8 vs 501.54 ± 124.95, *P* < .01), respectively. However, there were no adverse events resulted from prolonged withdrawal time.

### 3.2. Polyp and ADR

The comparison of PDR, ADR and MAP between the CWP and CC groups is summarized in Table 3. A total of 536 patients had ≥ 1 polyp, with 304 patients (PDR: 84.9%) in the CWP group and 232 patients (PDR: 59.8%) in the CC group. PDR was significantly higher among the patients in the CWP group (*P* < .001). A total of 442 patients (59.2%) had ≥ 1 adenoma, with 251 patients (ADR: 70.1%) in the CWP group and 191 patients (ADR: 49.2%) in the CC group. ADR was significantly higher among the patients in the CWP group (*P* < .001). MAP was also significantly higher in the CWP group than in the CC group (1.69 ± 1.93 vs 1.06 ± 1.59, *P* < .001). Proximal MAP was significantly higher in the CWP group than in the CC group (1.24 ± 1.63 vs 0.55 ± 1.01, *P* < .001); however, distal MAP did not differ between the 2 groups (0.46 ± 0.78 vs 0.51 ± 1.02, *P* = .561). The comparison of MAP between the CWP and CC groups stratified by sex is summarized in Table 4. In both males and females, total and proximal MAP in the CWP group were significantly higher than those in the CC group, respectively. In contrast, distal MAP was not different between 2 groups for either sex.

**Table 2:** Baseline characteristics of the 2 groups.

	Total (n = 746)	CWP (n = 358)	CC (n = 388)	*P*-value
Sex, n (%)				
Male	333 (44.6)	165 (46.1)	168 (43.3)	.46
Female	413 (55.4)	193 (53.9)	220 (56.7)	
Age, yrs*	62.9 ± 7.8	62.4 ± 7.7	63.3 ± 7.9	.07
Indication, n (%)				.99
Screening	419 (56.1)	200 (55.9)	219 (56.4)	
Bowel symptoms	72 (9.7)	35 (9.8)	37 (9.5)	
Surveillance	153 (20.5)	72 (20.1)	81 (20.9)	
Positive FOBT	102 (13.7)	51 (14.2)	51 (13.2)	
Cecal intubation time, s*	480.8 ± 327.5	529.6 ± 367.4	432.3 ± 288.1	<.01
Withdrawal time, s*	829.8 ± 401.0	950.7 ± 438.5	718.1 ± 326.0	<.01
Withdrawal time when polyp detection is zero, s* (n)	526.7 ± 135.5 (n = 210)	599.4 ± 139.8 (n = 54)	501.54 ± 124.95 (n = 156)	<.01
Bowel preparation (Aronchick scale), n (%)				.09
Fair	42 (5.6)	14 (3.9)	28 (7.2)	
Good	131 (17.6)	59 (16.5)	72 (18.6)	
Excellent	573 (76.8)	285 (79.6)	288 (74.2)	

* Age, cecal intubation time, and withdrawal time are represented as mean ± standard deviation.

CC = conventional colonoscopy, CWP = combining modified cap-assisted and water-exchange colonoscopy with prone position, FOBT = fecal occult blood test.

**Table 3:** Comparison of polyp/ADR and the MAP between 2 groups.

	CWP (n = 358)	CC (n = 388)	*P*-value
PDR, n (%)			
Total	304 (84.9)	232 (59.8)	<.01
Female	152 (78.7)	117 (53.2)	<.01
Male	152 (92.1)	115 (68.4)	<.01
ADR, n (%)			
Total	251 (70.1)	191 (49.2)	<.01
Female	116 (60.1)	89 (40.4)	<.01
Male	135 (82.8)	102 (60.7)	<.01
MAP, n*			
Total	1.69 ± 1.93	1.06 ± 1.59	<.01
Proximal	1.24 ± 1.63	0.55 ± 1.01	<.01
Distal	0.46 ± 0.78	0.51 ± 1.02	.56

* MAP is represented as mean ± standard deviation (range).

ADR = adenoma detection rate, CC = conventional colonoscopy, CWP = combining modified cap-assisted and water-exchange colonoscopy with prone position, MAP = mean number of adenomas detected per procedure, PDR = polyp detection rate.

**Table 4:** Comparison of the MAP between 2 groups, by sex.

MAP, n*	CWP	CC	*P*-value
Male			
Total	2.28 ± 2.24	1.49 ± 1.92	<.01
Proximal	1.73 ± 1.98	0.74 ± 1.21	<.01
Distal	0.57 ± 0.86	0.74 ± 1.23	.53
Female			
Total	1.19 ± 1.45	0.73 ± 1.18	<.01
Proximal	0.83 ± 1.11	0.40 ± 0.78	<.01
Distal	0.36 ± 0.68	0.33 ± 0.78	.19

* MAP is represented as mean ± standard deviation (range).

CC = conventional colonoscopy, CWP = combining modified cap-assisted and water-exchange colonoscopy with prone position, MAP = mean number of adenomas detected per procedure.

## 4. Discussion and conclusions

Screening colonoscopy is used widely in the prevention of colorectal cancer. Among the quality indicators governing the efficacy of colonoscopy, ADR and MAP are important indicators that may lead to a decrease in the rate of interval cancer.^[9,11]^ Although several methods, such as Endocuff, Endocuff-Vision, or Endorings, have been studied for these 2 quality indicators^[15]^; in countries with a National Health Insurance System, such as in Korea, there are limitations in adopting these methods for private clinics. Therefore, it is important to determine whether ADR can be increased using conventional methods.

The combination colonoscopies, including CWP, was effective in the detection of polyps and adenomas, especially those located in the proximal colon. These results may have been associated with the synergistic or complementary effects during the insertion and withdrawal phases.

During the insertion (water exchange) phase, the transparent hood prevents the occlusion of the suction channel by creating a space between the mucosa and the suction port; besides, the continuous water exchange prevents the settlement of debris onto the cap attachment. This interaction enables uninterrupted water exchange and a clear visualization during the insertion phase. Whereas, combination colonoscopy induces a synergistic effect, resulting in the prevention of loop formation and facilitation of cecal intubation by the following mechanisms: the cap-assisted colonoscopy provides better visualization of the lumen in the colonic flexures and the sigmoid colon, facilitating the advancement of the endoscope without forming excessive loops and inadequate air insufflation,^[21]^ the water-exchange colonoscopy minimizes the colonic distension, facilitating the advancement of the endoscope without forming excessive loops, prone positioning, owing to the patient’s body weight, provides generalized abdominal pressure; it may allow the passage of the colonoscope in specific instances, such as when large loops, that are otherwise challenging to resolve, form in the transverse colon.^[16,22]^ While these effects may not facilitate the effective inspection of the mucosa directly, they enable the movement of the colonoscopic tip and allows the endoscopist to concentrate, without exhaustion, particularly while inspecting the mucosa during the withdrawal phase.

**Figure 2.**
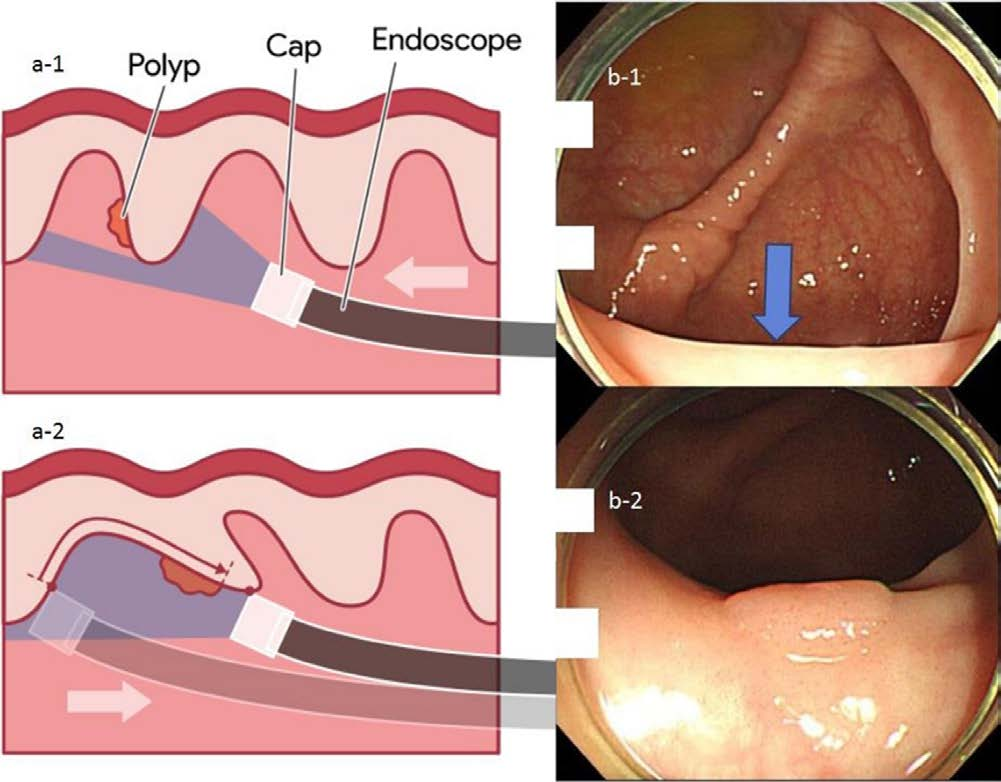
“Hooking and dragging maneuver” revealing hidden polyp. The “a-1” and “b-1” shows the view without the “hooking and dragging maneuver”. In this view, the polyp, which is at the proximal side of the mucosal fold, is hidden. However, when ① hooking (gentle bending of the tip of the colonoscope towards the wall of the colon) and ② dragging (gentle retraction of the colonoscope dragging the multiple folds of the colon) is performed (a-2), the hidden polyp at the proximal side of the mucosal fold becomes visible (b-2). The arrowhead (b-1) indicates the position where the polyp was hidden.

**Figure 3.**
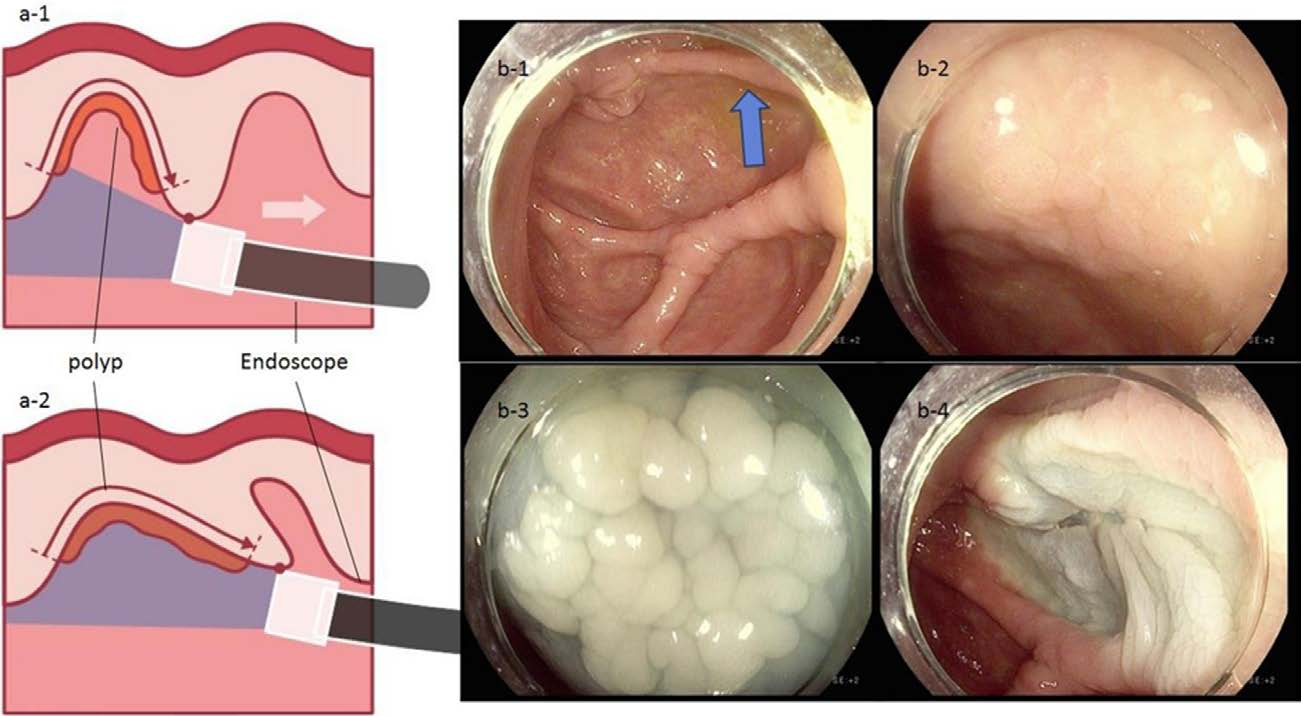
“Hooking and dragging maneuver” revealing the LST, hidden between mucosal folds. The “a-1” and “b-1” shows the view without the “hooking and dragging maneuver”. In this view, LST, which is between mucosal folds, is hidden. However, when hooking and dragging is performed (a-2), the hidden LST between the mucosal folds becomes visible (b-2). The arrowhead (b-1) indicates the position where LST was hidden. The submucosal injection is being administered (b-3), and the EMR is being performed (b-4). EMR = endoscopic mucosal resection, LST = lateral spreading tumor.

**Figure 4.**
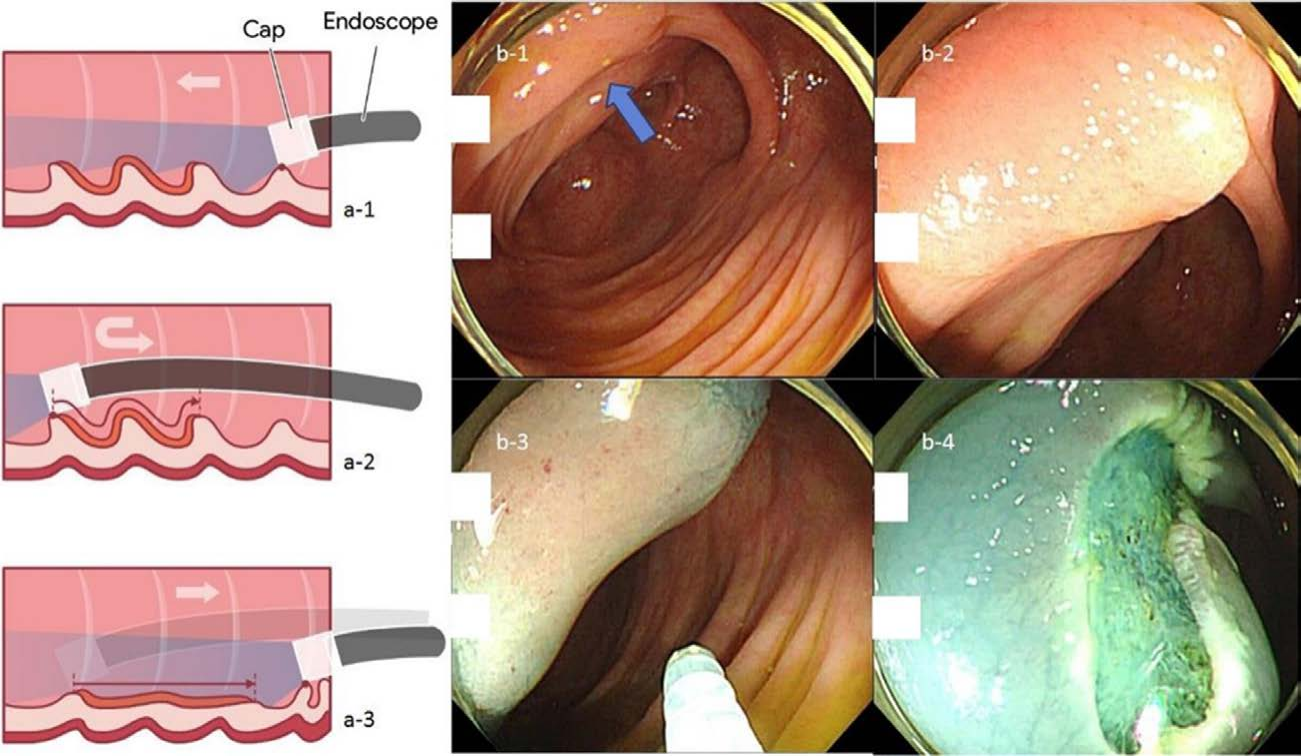
“Hooking and dragging maneuver” revealing hidden LST, overlying multiple mucosal folds. The “a-1” and “b-1” shows the view without the “hooking and dragging maneuver”. In this view, the LST, overlying multiple mucosal folds, is hidden. However, when hooking (a-2) and dragging (a-3) is performed, the hidden LST overlying the multiple mucosal fold becomes visible (b-2). The arrowhead indicates the position where LST was hidden (b-1). The submucosal injection is being administered (b-3), and the EMR is being performed (b-4). EMR = endoscopic mucosal resection, LST = lateral spreading tumor.

Improved quality of cleanliness on water-exchange colonoscopy^[13]^ and better visualization of proximal side of mucosal folds on cap-assisted colonoscopy^[23–25]^ may explain why proximal MAP, but not distal MAP, showed a significant increase in the CWP group. We also used a modified version of the cap-assisted colonoscopy, referred to as the “hooking and dragging maneuver”.^[20]^ The conventional cap-assisted colonoscopy flattened a single mucosal fold,^[26]^ while the modified cap-assisted colonoscopy hooked (gentle bending of the tip of the colonoscope towards the wall of the colon) and dragged (gentle retraction of the colonoscope, dragging the multiple folds of the colon) multiple mucosal folds simultaneously.^[20]^ This modified method simultaneously allowed the inspection of the proximal and distal parts of the mucosal folds, minimizing blind spots. Figures 2, 3 and 4 demonstrate the use of the hooking and dragging maneuver in locating hidden polyps in multiple cases, correlated with real-world cases. Even though a recent meta-analysis study showed both water exchange [1.46 (1.20–1.76)] and Endocuff [1.39 (1.17–1.66)] increased ADR, while cap assisted colonoscopy has no impact on ADR [1.00 (0.82–1.22)],^[27]^ an important thing has been overlooked. In cap-assisted colonoscopy, no attempt was made to standardize the usage of the cap, and it is likely that non-standardized usage influenced the results of the analysis. A study about artificial intelligence identifying blind spots showed that a single endoscopist with an ADR of 47% missed 1% to 50% of the total colon surface area (median 19%).^[28]^ Using the cap only for insertion as well as for “hooking and dragging maneuver” is likely to decrease the potential for a blind spot. Thus, it is necessary to reanalyze ADR and blind spots according to the cap usage maneuver and standardize it. In this study, ADR in the CWP group (70.1%) were higher than that of previous studies with 40.5% in cap-assisted colonoscopies (meta-analysis),^[29]^ 41.7% in water exchange colonoscopies (network meta-analysis),^[13]^ and 44% in cap-assisted water immersions (single-center trial).^[30]^ High ADR in this study could be associated with the high quality and long withdrawal time of CWP. The significantly longer withdrawal time in the CWP group compared to the CC group was not only due to the additional time required for subsequent polypectomies that resulted from high detection rates but also because of the “hooking and dragging maneuver” in the modified cap-assisted colonoscopy. Compared to no polyp detected cases, withdrawal time is also significantly longer in the CWP group (599.46 ± 139.85 [n = 54] vs 501.54 ± 124.95 [n = 156], *P* < .001). In addition, comparing PDR, ADR and MAP between males and females, males were significantly higher than females in total patients, the CWP and the CC groups. These findings were consistent in that of previous studies.^[31,32]^ Although exact pathophysiologic mechanisms are still unknown, the differences of levels of hormones, smoking rates, metabolic syndrome, and lifestyle may be the associated factors for sex differences.^[33,34]^

According to the study by Yen et al,^[10]^ investigating the combined method without the prone position, ADR for the combination of the water-exchange and cap-assisted colonoscopies was 75% (PDR, 93.0%; proximal colon ADR, 61%; adenoma per colonoscopy, 2.70) and was consistent with ADR of this study. However, MAP (1.69 ± 1.93) of this study was lower than MAP (2.70 ± 3.27) obtained in their study.^[10]^ Considering that most of the participants in the study by Yen et al were males (male, 95; female, 5), we evaluated PDR, ADR, MAP and proximal MAP according to sex. In this study, the PDR, ADR, and MAP in males were 92.1%, 82.8%, and 2.28 ± 2.24, respectively. High ADR in both the study by Yen et al and this study demonstrated that the combination of cap-assisted and water-exchange colonoscopies facilitated the detection of adenomas by the aforementioned synergistic or complementary effects. Yen et al used Olympus PCF-H180AL colonoscope (Olympus Medical Systems Corp. Shinjuku City, Tokyo, Japan), with variable stiffness capability, in patients without changing their posture to the prone position, however, the prone position can be helpful while using a colonoscope without variable stiffness capability.

This study had some limitations. First, this was a retrospective study and was performed in a single center; hence, the potential selection or information bias may have existed. Second, we could not investigate the patients’ family history of colorectal cancer smoking history, stool frequency and stool property, which could have influenced ADR,^[35,36]^ and body mass index, which could have led to an increase in loop formation.^[37]^ Third, the endoscopists were non-blinded to the methods used in this study, which may have been a potential source of investigator bias. Fourth, only 2 of the endoscopists participated in this study, and they performed only 1 method according to their skill proficiency. Fifth, the benefit of prone position in combined colonoscopy was unclear. Therefore, further evaluation such as a prospective, multicenter study using fluoroscopy needed to prove the role of prone position in colonoscopy.

In conclusion, ADR and MAP in the CWP group were significantly higher than that in the CC group, suggesting that CWP could be more useful than CC. Although this technique can improve adenoma detection, further evaluation to assess these synergistic effects is required.

## Supplementary Material


